# Oculomotor Nerve Palsy Secondary to Cavernous Internal Carotid Aneurysm

**DOI:** 10.5811/cpcem.2017.10.35510

**Published:** 2018-01-09

**Authors:** Gary Lai, Maria I. Rodriguez, Alexander J. Scumpia

**Affiliations:** * Broward Health, Coral Springs Hospital, Department of Emergency Medicine, Coral Springs, Florida; † Broward Health, Imperial Point Hospital, Department of Emergency Medicine, Fort Lauderdale, Florida

## CASE PRESENTATION

**Image 1:**
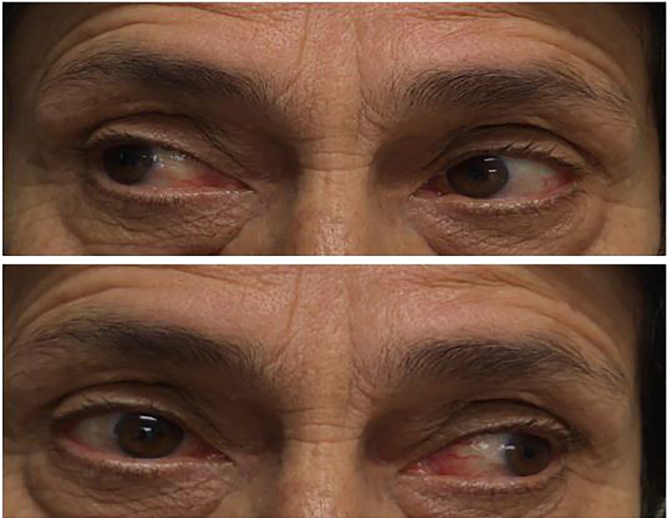
Rightward gaze demonstrating normal ocular movements (top image). Leftward gaze demonstrating absent right ocular abduction, illustrating a third nerve palsy (bottom image)

**Image 2:**
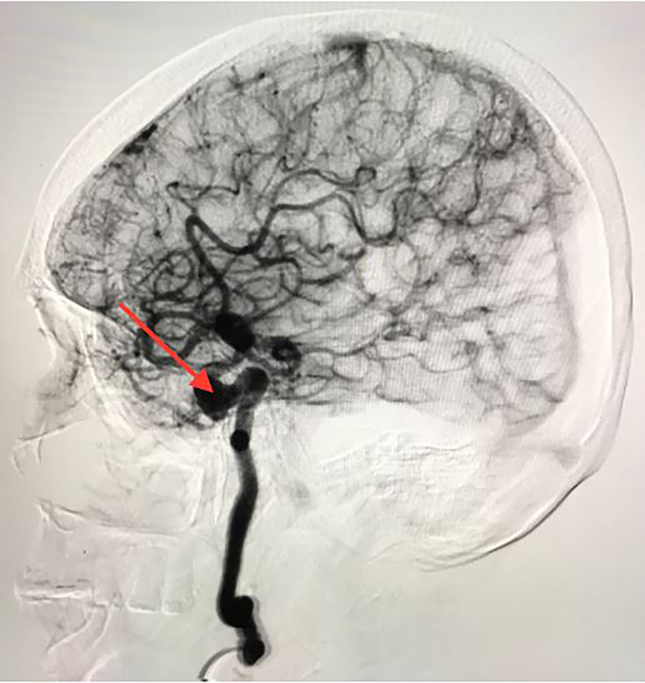
Sagittal cerebral angiogram demonstrating the right fusiform, 9 × 7.5-millimeter cavernous internal carotid aneurysm (red arrow)

A 68-year-old female presented to the emergency department with progressively worsening, atraumatic right-eye blurred vision, dull headache and mild nausea over the preceding two days. Her daughter also noticed that the patient’s right eye was displaced inferolaterally or “down and out.” The patient denied photophobia, neck stiffness, rash, myalgias, or changes in speech or gait. Vital signs and laboratory data were unremarkable. After a neurological examination raised the suspicion of intracranial pathology (Image 1) appropriate radiographic imaging was ordered. The lesion was confirmed via computed tomography angiography with subsequent cerebral angiography (Image 2) demonstrating a 9 × 7.5 millimeter cavernous internal carotid aneurysm.

## DISCUSSION

Oculomotor nerve palsy has been classically separated into pupil sparing and non-pupil sparing (i.e., pupils that react to light). Common causes for pupil-sparing pathologies are diabetic neuropathy, myasthenia gravis, atherosclerosis, chronic progressive opthalmoplegia and vasculopathies (i.e., giant cell arteritis and temporal arteritis).^1^ The accepted pathophysiological mechanism of this phenomenon is the formation of vascular lesions occluding the vaso-nervorum leading to ischemic infarction, sparing the parasympathetic fibers located peripherally of the third cranial nerve (62–83% of cases).^1^ On the other hand, the most common causes of non-pupil sparing oculomotor palsy are tumor (i.e., chordomas, clival meningiomas), followed by vascular lesions (posterior communicating aneurysms,^2^ and then distal basilar artery aneurysms). Even rarer presentations are uncal herniation and, least commonly (5%),^1^ cavernous sinus lesions (including tumor, vascular pathologies).

Cavernous sinus syndrome from lesions can cause multiple nerve palsies due to the anatomical constituents of the oculomotor (III), trochlear (IV), trigeminal ophthalmic and maxillary divisions (V1 andV2) and abducens (VI).^3^ Third nerve palsy secondary to cavernous internal carotid aneurysms will not produce a dilated pupil, since sympathetic fibers that cause dilatation are also paralyzed.^4^ This was true to the case described herein where the patient’s right pupil was not “blown.” This case illustrates the complexity of the cavernous sinus and the utilization of computed tomography angiography to achieve appropriate clinical diagnosis. The patient ultimately underwent successful neuro-endovascular treatment and was subsequently discharged five days later.

CPC-EM Capsule

What do we know about this clinical entity?

Many previously reported intracranial pathologies can cause oculomotor nerve palsy, including endocrine, aneurysms, and tumors.

What is the major impact of the image(s)?

Cavernous sinus internal carotid aneurysms, although rare, can cause oculomotor nerve palsy. The images demonstrate the necessity for advanced imaging, which is essential for proper diagnosis.

How might this improve emergency medicine practice?

The neuroanatomical complexity of the cavernous sinus should raise the emergency physician’s suspicion for intracranial lesions.
